# The impact of different gastrointestinal reconstruction techniques on gut microbiota after gastric cancer surgery

**DOI:** 10.3389/fmicb.2024.1494049

**Published:** 2025-01-24

**Authors:** Yu Yang, Hang-Yu Zhou, Guo-Min Zhou, Jin Chen, Rui Ming, Dong Zhang, Huai-Wu Jiang

**Affiliations:** ^1^Department of Gastrointestinal Surgery, The Affiliated Hospital of Southwest Medical University, Luzhou, Sichuan, China; ^2^Department of Gastrointestinal Surgery, Mianyang 404 Hospital, Mianyang, Sichuan, China

**Keywords:** gastric cancer, gut microbiota, 16S rRNA sequencing, double tract reconstruction, Roux-en-Y gastrointestinal reconstruction

## Abstract

**Introduction:**

Gastric cancer is one of the common malignant tumors in the digestive tract, characterized by high incidence and mortality rates. This is particularly significant in China, where a large proportion of global new cases of gastric cancer and related deaths occur. In recent years, with the continuous development of molecular biology technology, people have gained a deeper understanding of the gastrointestinal microbiome, and studies have shown that it is closely related to the occurrence, development, and therapeutic response of gastric cancer. Although surgical intervention is crucial in significantly extending the survival of gastric cancer patients, the disruption of the balance of the intestinal microbiota caused by surgery itself should not be overlooked, as it may affect postoperative recovery.

**Methods:**

This study was approved by the Biomedical Ethics Committee of Sichuan Mianyang 404 Hospital. A random sampling method was used to select patients who underwent gastric cancer surgery at the hospital from January 2023 to December 2023. All patients signed written informed consent forms. Standardized perioperative management was conducted for the patients in the study, including preoperative preparation, intraoperative handling, and postoperative treatment. Fecal samples were collected from patients before surgery (before bowel preparation) and around one week after surgery for 16S rRNA sequencing analysis, through which differential biomarkers and related functional genes were sought.

**Results:**

The study results indicated that there was no significant difference in the diversity of the gut microbiota between the two groups. Compared with the R-Y group, the DTR surgical method significantly altered the structure of the gut microbiota, affecting the types, quantities, and proportions of intestinal bacteria. Furthermore, the DTR group exhibited poorer postoperative nutritional absorption capacity compared to the R-Y group, as indicated by a lower F/B ratio. The R-Y group showed a richer abundance of Bacteroidetes and a lower abundance of Proteobacteria, as well as a higher F/B ratio after surgery. These findings provide new insights into the changes in the gut microbiota following gastric cancer surgery, which may be of significant importance for postoperative recovery and long-term health management.

**Discussion:**

This study reveals the impact of different gastrointestinal reconstruction techniques on the postoperative gut microbiota of gastric cancer patients, providing new insights into the physiological changes during the postoperative recovery period. Although there was no significant difference in microbial diversity between the DTR group and the R-Y group, the DTR group showed more pronounced changes in microbial structure postoperatively, which may be associated with an increased risk of postoperative infection. These findings emphasize the importance of considering the impact on the gut microbiota when selecting gastric cancer surgery methods. However, the study had a limited sample size and did not delve into changes in metabolites. Future studies should expand the sample size and conduct metabolomic analyses to further validate these preliminary findings.

## 1 Introduction

Gastric cancer, a common malignant tumor in the digestive tract, is marked by high incidence and mortality rates. According to statistical data from the International Agency for Research on Cancer (IARC) (Pizzato et al., 2022), there are as many as 1.089 million new cases of gastric cancer globally each year, with 769,000 deaths, ranking it fifth among all malignant tumors in terms of incidence and fourth in mortality. It is noteworthy that the incidence and mortality of gastric cancer are particularly prominent in China. Approximately 42.6% of global gastric cancer incidence and 45% of gastric cancer-related deaths occur in China (GBD 2017 Stomach Cancer Collaborators, 2020), closely linked to the low rates of early gastric cancer screening and diagnosis in the country. Early detection of precancerous lesions of gastric cancer (PLGC) and prevention of their progression are crucial for reducing the incidence of gastric cancer.

In recent years, the gastrointestinal microbiome has become a hot topic of research. With the continuous development of molecular biology techniques, 16S rRNA technology, metagenomic research, and high-throughput sequencing technology, we have gained a deeper understanding of the gastric microbiota. These technologies provide strong support for the study of gastric microecology. Research indicates that the gastrointestinal microbiota accounts for about 76% of the human microbiota (Moossavi and Azad, 2019), including a variety of microorganisms such as bacteria, viruses, and fungi, among which bacteria play a dominant role, together forming a complex microbial ecosystem (Laukens et al., 2016). It is worth mentioning that recent breakthroughs have been made in the study of the pathogenesis of gastric cancer, with a growing body of evidence pointing to a close relationship between gastric cancer and the dysregulation of bacteria in the stomach, especially the infection of *Helicobacter pylori* (H. pylori). This bacterium can cause chronic gastritis and gastric ulcers, and may also induce malignant transformation of gastric mucosal cells under long-term chronic stimulation (GBD 2017 Stomach Cancer Collaborators, 2020). At the same time, the gut microbiota also has an important impact on the occurrence, development, and therapeutic response of gastric cancer (Kubota et al., 2014; Kanda et al., 2019).

Although surgical intervention is pivotal in significantly prolonging the survival of gastric cancer patients, the iatrogenic trauma inherent in the procedure exerts a non-negligible effect on the equilibrium of the gut microbiota. Empirical evidence suggests that the stress induced by surgical trauma can modulate the permeability of the intestinal mucosa postoperatively, precipitating dysbiosis, which in turn amplifies intestinal inflammatory responses, compromises the immune competence of the patient, and thereby directly impedes the trajectory of postoperative convalescence (Hyoung et al., 2018). Moreover, the inflammatory cascade initiated by gastric cancer surgery leads to a marked escalation in the levels of interleukin-6 (IL-6) and tumor necrosis factor-alpha (TNF-α), concurrent with a diminution in the expression of immunoregulatory cytokines. The concomitant impairment of the intestinal mucosal barrier facilitates the translocation of enteric bacteria, exacerbating enteric inflammation, thereby establishing a deleterious feedback loop (Sánchez-Zauco et al., 2017; Muñoz et al., 2019).

The changes in the gut microbiota caused by surgery have profound effects on the health and postoperative recovery of patients, involving digestion, immune regulation, metabolism, and more (Kau et al., 2011; Pflughoeft and Versalovic, 2012; Shreiner et al., 2015). In terms of digestion and nutrient absorption, the gut microbiota plays a crucial role, helping to break down dietary fiber, synthesize vitamins such as vitamin K and B vitamins, and promote the absorption of minerals. After gastric cancer surgery, changes in the gut microbiota may lead to indigestion and poor nutrient absorption, affecting the patient’s nutritional status and recovery. Disruption of the gut microbiota can affect the absorption of vitamin B12 (Degnan et al., 2014; Jun et al., 2016), thereby exacerbating the patient’s nutritional intolerance and anemia symptoms, delaying recovery (Radigan, 2004; Lim et al., 2019). The gut microbiota is also involved in various metabolic pathways, including energy metabolism and lipid metabolism, affecting fat storage and distribution, as well as sugar metabolism, thereby affecting the patient’s weight and blood sugar control. Changes in the gut microbiota after surgery may affect these metabolic pathways, increasing the risk of developing metabolic syndrome and type 2 diabetes (Turnbaugh et al., 2006; Turnbaugh et al., 2009; Karlsson et al., 2013). In addition, the gut microbiota is closely related to the host’s immune system, and imbalances in the gut microbiota after surgery may lead to decreased immune function, increasing the risk of infection (Rubinstein et al., 2013; Kasai et al., 2016; Yachida et al., 2019).

In the clinical management of gastric cancer, radical surgery is regarded as the most efficacious therapeutic intervention, which includes the extirpation of the tumor, lymph node dissection, and the critical phase of gastrointestinal tract reconstruction. The latter is paramount for the convalescence of patients (Yan et al., 2022; Wu et al., 2023). The reconstruction of the gastrointestinal tract post-gastrectomy is an essential procedure in clinical therapy and has become a central focus within the medical community (Zhou et al., 2020). Currently, the treatment paradigm for gastric cancer is predominantly surgical, complemented by a comprehensive strategy that integrates a multi-disciplinary team (MDT) approach, which is crucial for the holistic management of gastric cancer (Gastric Cancer MDT Expert Consensus, 2017). Gastric cancer predominantly arises in the antral region, and the standard surgical protocol involves a distal gastrectomy accompanied by a D2 lymph node dissection. Post-distal gastrectomy, the reconstruction of the gastrointestinal tract commonly employs techniques such as the Billroth I and II anastomoses, the Roux-en-Y (R-Y) anastomosis, and its variant, the Uncut R-Y Approaches.

Double-tract reconstruction (DTR) is a method of gastrointestinal reconstruction used in gastric cancer surgery, especially after proximal gastrectomy. This technique aims to reduce food reflux and maintain the normal digestive pathway by creating two channels (Chen et al., 2017; Long et al., 2018). In DTR, an esophagojejunal R-Y anastomosis is first performed, followed by a lateral-to-lateral anastomosis between the distal residual stomach and the jejunum 10–15 centimeters away from the esophagojejunal anastomosis, allowing food to enter the distal jejunum through two channels. Its advantages include good anti-reflux effects and less demanding requirements for the residual stomach. Despite the presence of two pathways, this reconstruction method has advantages for the absorption of micronutrients such as vitamin B12 and iron (Kohzadi et al., 2017; Zoroddu et al., 2019). Studies have shown that compared with traditional R-Y anastomosis, DTR can more quickly restore gastrointestinal function (Qiu et al., 2022; Ying et al., 2023). However, there is less research on the impact of postoperative gut microbiota, and this study aims to analyze the effects of different surgical methods on the gut microbiota.

This study delved into the impact of different gastrointestinal reconstruction techniques on the gut microbiota of patients following gastric cancer surgery, which holds significant importance for understanding the complex physiological changes during postoperative recovery. By comparing the DTR with the R-Y gastrointestinal reconstruction, the study revealed the significant effects of different digestive tract reconstruction methods on the diversity and structure of the gut microbiota. These findings not only provide valuable references for the clinical treatment of gastric cancer surgery but also offer a scientific basis for developing targeted postoperative management strategies, such as nutritional support and infection control.

## 2 Materials and methods

### 2.1 Study design

This study was approved by the Biomedical Ethics Committee of Sichuan Mianyang 404 Hospital (Trial No. 028 in 2022). All study patients signed written informed consent. A randomized sampling method was used to select gastric cancer patients who underwent surgical treatment at Sichuan Mianyang 404 Hospital from January 2023 to December 2023 as subjects of the study.

**FIGURE 1:**
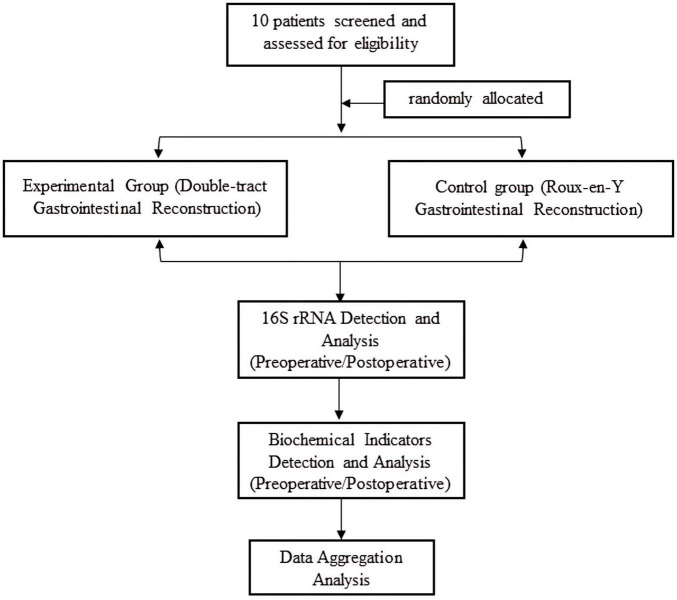
Trial profile.

The trial profile is shown in Figure 1. Inclusion criteria: (1) Patients diagnosed with gastric cancer by pathology; (2) Patients with locally advanced gastric cancer scheduled for radical resection; (3) Age ≤ 75 years, both genders included; (4) Performance status score: KPS ≥ 80; (5) Willing to participate and have signed the informed consent form; (6) No distant organ metastasis on preoperative examination, and no prior radiotherapy or chemotherapy. Exclusion criteria: (1) Early-stage gastric cancer; (2) Distant metastasis present; (3) Performance status score: KPS < 80, or overall condition unable to tolerate surgery; (4) History of intestinal surgery, cholecystectomy, or pancreas surgery; (5) History of functional dyspepsia; (6) Severe cardiac, pulmonary, hepatic, or renal insufficiency; (7) Infectious diseases present, or other conditions affecting quality of life; (8) Inability to understand or refusal to sign the informed consent form. Disqualification criteria: (1) Violation of entry criteria; (2) Failure to complete surgery as planned; (3) Missing main indicators, with obviously incomplete data. Withdrawal criteria: (1) Patient’s own request to withdraw from the trial; (2) Emergence of other serious medical conditions deemed unrelated to gastrointestinal reconstruction surgery by the investigator; (3) Inability to follow the protocol for follow-up, poor compliance; (4) Emergence of other serious medical conditions preventing completion of follow-up observations.

### 2.2 Perioperative management

The perioperative management protocol for both cohorts of patients was standardized, encompassing the following measures. Preoperative Preparation Phase: (1) For patients without gastrointestinal motility disorders, they must refrain from consuming any food within 6 h prior to surgery, and within 2 h before the commencement of the surgery, they must completely cease the intake of any liquids. (2) To avoid unnecessary intervention before the surgery, no gastric tube will be placed for the patients. (3) Considering the balance of the intestinal microbiota, within 3 months prior to surgery, patients should not receive any treatments or medications that may affect this balance, such as probiotics, antibiotics, and proton pump inhibitors. Intraoperative treatment: (1) All surgeries are performed by the same medical team. (2) General anesthesia is administered in all cases. (3) Patients undergo either proximal subtotal gastrectomy or total gastrectomy. (4) The Approaches of gastrointestinal tract reconstruction include R-Y reconstruction or the modified DTR gastrointestinal reconstruction. (5) Prophylactic administration of ceftriaxone 2 grams via intravenous drip is carried out 30 min before surgery. Postoperative treatment: (1) A nasogastric tube is routinely left in place after surgery, typically for a period of 5–6 days. (2) After the removal of the nasogastric tube, patients begin with clear fluids and progress to semi-solid foods on the second day following tube removal. (3) Administer cefuroxime intravenously every 12 h (q12h) at a dosage of 1.5g, or ceftriaxone once daily (qd) at a dosage of 2 grams. (4) Probiotics or other live bacterial preparations are not used before discharge after surgery. (5) All patients receive and follow the dietary guidance provided by our hospital’s nutrition department postoperatively.

### 2.3 Sample collection

Preoperatively (before bowel preparation), blood and fecal samples are collected from patients. A fecal microbiota analysis is conducted on the fresh, medium-to-end segment, internal 2 g fecal samples. The samples are named RQ (preoperative samples of the R-Y group), SQ (preoperative samples of the DTR group), and are immediately frozen at −80°C for preservation. After all patients have completed the relevant preoperative preparations, they undergo surgical treatment under general anesthesia. Postoperatively, they are all given ceftriaxone (1 g) intravenous infusion every 12 h for infection prevention, which is generally used for 48 h and then discontinued. On the first postoperative day, parenteral nutritional support is initiated for all patients. After the patients resume a liquid diet (porridge or milk) and have a bowel movement of yellow stool, blood and fecal samples are collected again (approximately 1 week postoperatively). The fecal samples are named RH (postoperative samples of the R-Y group), SH (postoperative samples of the DTR group), consistent with the preoperative samples and preserved under the same low-temperature conditions. After all specimens are collected, they are sent together to Beijing Biomarker Technologies Co., LTD. (Beijing, China) for testing and analysis.

### 2.4 Microbiome analysis

Firstly, the raw reads obtained from sequencing were filtered using the Trimmomatic v0.33 software. Subsequently, the Cutadapt 1.9.1 software was utilized to identify and remove primer sequences, yielding clean reads free of primer sequences. The reads were then processed with the dada2 method in QIIME2 2020.6 (Bolyen et al., 2019) for denoising, merging of paired-end sequences, and the removal of chimeric sequences, resulting in the final valid data (Non-chimeric Reads). Finally, differential biomarkers were sought, and related functional genes were predicted through analyses such as alpha diversity analysis, beta diversity analysis, inter-group difference significance analysis, and functional gene prediction (Kenneth et al., 1975; Ramette, 2007).

### 2.5 Statistical analysis

The data were processed and analyzed using the R software package. For comparisons between two groups, t-test analysis was utilized, while non-parametric rank sum tests were performed using the Kruskal-Wallis (H) test and the Wilcoxon test. A *p*-value of less than 0.05 was considered to indicate statistical significance, and a *p*-value of less than 0.01 was regarded as indicating a high level of statistical significance.

## 3 Results

A total of 20 fecal samples were collected (one serving as a normal control sample), with 5 patients in each of the R-Y surgery group and the DTR surgery group. Patient information and perioperative and postoperative test indicators for the two groups are shown in Table 1 (all *p* > 0.05).

**TABLE 1:** Baseline characteristics and outcomes of patients with different surgical approaches.

Parameter	R-Y surgery group (*n* = 5)	DTR surgery group (*n* = 5)	*P*
Age, years mean ± SD	65.2 ± 3.11	68 ± 4.36	0.280
Body mass index kg/m^2^, mean ± SD	23.30 ± 4.52	23.41 ± 2.83	0.893
Intraoperative blood loss ml, mean ± SD	18 ± 4.47	26 ± 13.42	0.263
Surgical duration hours, mean ± SD	3.53 ± 0.43	3.4 ± 0.84	0.671
Degree of total protein decrease (TP) g/L, mean ± SD	11.14 ± 9.23	19.74 ± 4.04	0.109
Degree of serum Albumin decrease (ALB) g/L, mean ± SD	6.1 ± 5.16	11.28 ± 3.73	0.110
Degree of Hemoglobin decrease (Hb) g/L, mean ± SD	4.6 ± 5.13	17.8 ± 14.17	0.107
Degree of Leukocyte increase(WBC) g/L, mean ± SD	7.32 ± 5.94	4.46 ± 1.99	0.355
Degree of Neutrophil increase(Neu) g/L, mean ± SD	16.42 ± 17.23	12.58 ± 5.86	0.657

Statistical test performed: two-sample *t*-test for numerical data; *n*, number of participants.

### 3.1 16S rRNA sequencing

#### 3.1.1 Comparison of α-diversity after different surgical approaches

α-diversity, a key measure in ecological studies, reflects the species abundance and diversity within a single sample. The Chao1 index and Ace index are used to assess the number of species, while the Shannon index and Simpson index evaluate the diversity of species. The PD whole tree index is a phylogenetic diversity index calculated from the representative sequences of operational taxonomic units (OTUs) in each sample to construct a phylogenetic tree. The sum of the branch lengths of all representative sequences in a sample yields a value that indicates the community’s diversity, with higher values suggesting greater diversity.

**FIGURE 2:**
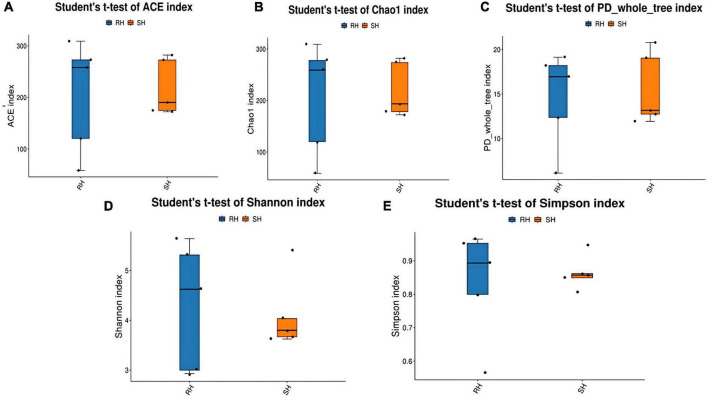
Comparison of postoperative α-diversity of gut microbiota among different surgical approaches. **(A)** ACE index; **(B)** Chao1 index; **(C)** PD whole tree index; **(D)** Shannon index; **(E)** Simpson index.

The t-test revealed that the R-Y surgery group had a higher level of species diversity and richness compared to the DTR surgery group. However, no significant differences were found between the indices (all *p*-values > 0.05), as shown in Figure 2. Additionally, we analyzed the α-diversity before and after different surgical approaches. There was no significant difference in the R-Y surgery group (Supplementary Figure 2), while the DTR surgery group showed significant differences in the Chao1 index, Ace index, and PD whole tree (Supplementary Figure 1).

#### 3.1.2 Comparison of β-diversity after different surgical approaches

**FIGURE 3:**
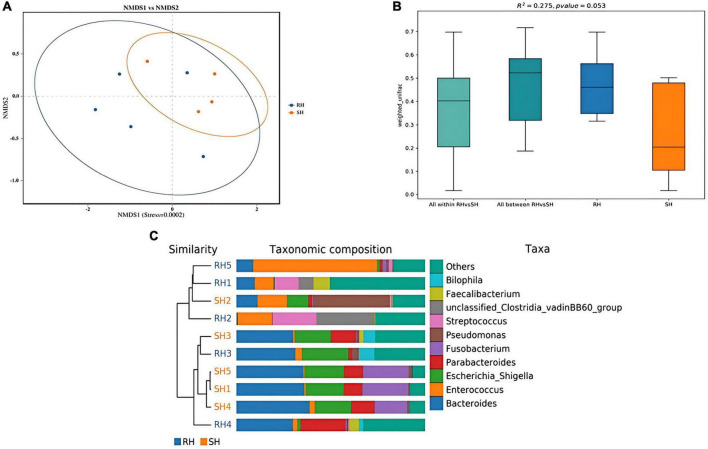
Analysis of postoperative gut microbiota β-diversity among different surgical approaches. **(A)** PCA analysis; **(B)** PerMANOVA analysis; **(C)** UPGMA clustering tree using weighted UniFrac distances.

β-diversity is primarily employed to assess the similarities in the composition of microbial communities across samples and to compare the levels of species diversity among different samples. The β-diversity of the gut microbiota in the R-Y surgery group and the DTR surgery group was analyzed using the NMDS algorithm with weighted UniFrac, as shown in Figure 3A. There is a certain degree of similarity in the microbial composition between the different surgical groups. The Adonis statistical analysis, based on the Bray-Curtis distance algorithm, revealed significant differences in β-diversity between the two groups (*p* = 0.053) (Figure 3B), indicating that different surgical Approaches can affect the structure of the gut microbiota (Figure 3C). In addition, we compared the changes in gut microbiota before and after different surgeries and found that the DTR surgery group could alter the structure of the gut microbiota (Supplementary Figure 3), while there was no significant change in the gut microbiota before and after the R-Y surgery group (Supplementary Figure 4).

#### 3.1.3 Influence of various surgical approaches on gut microbiota composition and differential microbiota analysis

**FIGURE 4:**
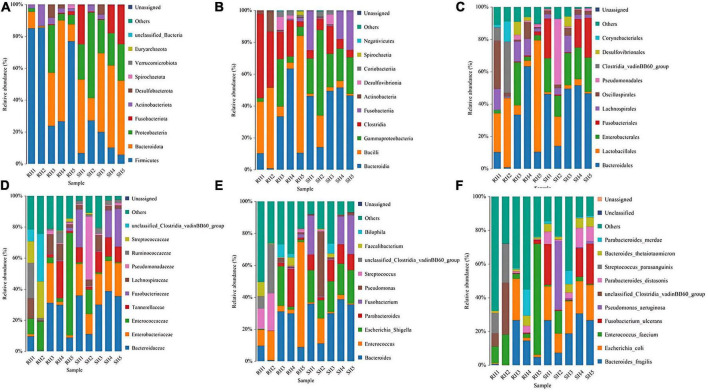
Phylogenetic analysis of relative abundances at different taxonomic levels for the two groups. **(A)** Phylum; **(B)** class; **(C)** order; **(D)** family; **(E)** genus; **(F)** species (display only the top 10 taxonomic groups by relative abundance at each classification level).

**FIGURE 5:**
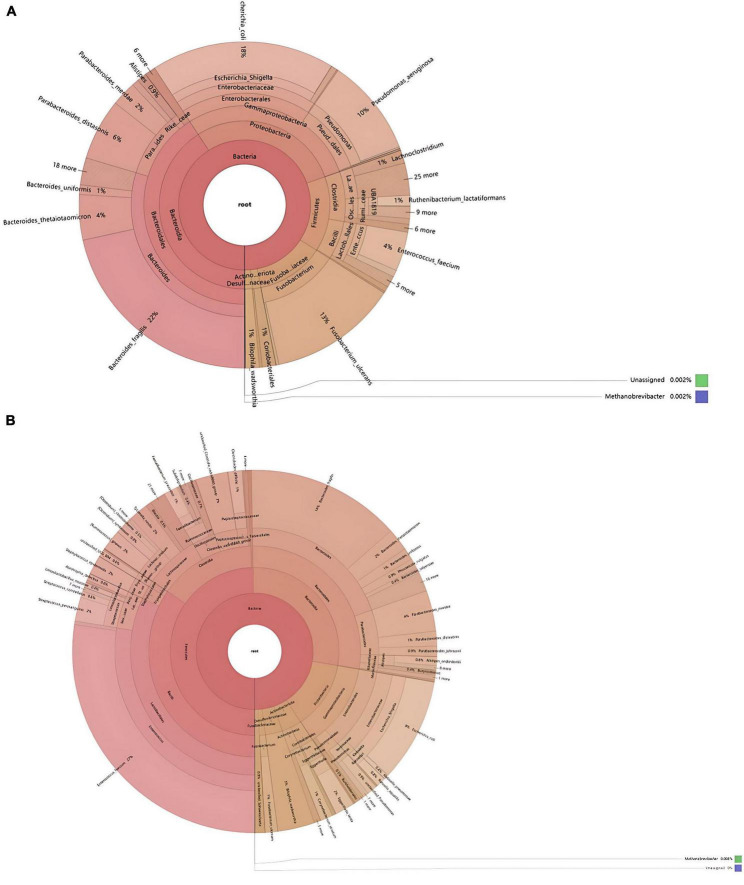
Comparative taxonomic composition of intestinal microbiota based on KRONA analysis. **(A)** SH; **(B)** RH (the circles from the inside out represent the five taxonomic levels of phylum, class, order, family, and genus, respectively, with the size of the sectors reflecting the relative abundance of different taxonomic units).

Through species annotation of sequencing data from samples, the dominant phyla, genera, and species annotated in the SH group are as follows: Phyla, Bacteroidia (41%), Proteobacteria (29%), Firmicutes (15%); Genera, Bacteroides (30%), Fusobacterium (13%), Pseudomonas (10%), Parabacteroides (9%), Enterococcus (5%), Lachnoclostridium (1%); Species, *Bacteroides fragilis* (22%), *Escherichia coli* (18%), *Escherichia Shigella* (18%), *Fusobacterium ulcerans* (13%), *Pseudomonas aeruginosa* (10%), *Parabacteroides distasonis* (6%), *Bacteroides thetaiotaomicron* (4%), *Enterococcus faecium* (4%), *Parabacteroides merdae* (2%). The F/B (Firmicutes/Bacteroidetes) ratio in the SH group is 0.37 (Figures 4, 5A).

For the RH group, the dominant phyla, genera, and species annotated are: Phyla, Bacteroidia (50%), Proteobacteria (13%), Firmicutes (28%); Genera, Enterococcus (28%), Bacteroides (20%), Parabacteroides (6%), Streptococcus (3%), Staphylococcus (2%), Lachnoclostridium (2%), Eggerthella (2%), Alistipes (1%), Corynebacterium (1%), Faecalibacterium (1%), Fusobacterium (1%); Species, *Enterococcus faecium* (27%), *Bacteroides fragilis* (14%), *Escherichia Shigella* (9%), *Escherichia coli* (9%), *Parabacteroides merdae* (4%), *Bilophila wadsworthia* (3%), *Bacteroides thetaiotaomicron* (2%), *Staphylococcus epidermidis* (2%), *[Ruminococcus] gnavus* (2%), *Tyzzerella nexilis* (2%), *Streptococcus parasanguinis* (2%), *Eggerthella lenta* (2%), *Parabacteroides distasonis* (1%), *Corynebacterium striatum* (1%), *Faecalibacterium prausnitzii* (1%), *Clostridioides difficile* (1%), *Bacteroides uniformis* (1%), *Fusobacterium ulcerans* (1%), *[Clostridium] symbiosum* (0.6%), *Streptococcus constellatus* (0.9%), *Parabacteroides johnsonii* (0.9%), *Phocaeicola vulgatus* (0.9%), *Alistipes onderdonkii* (0.8%), *Rahnella aquatilis* (0.8%), *Abiotrophia defectiva* (0.6%). The F/B (Firmicutes/Bacteroidetes) ratio in the RH group is 0.56 (Figures 4, 5B).

LEfse (Linear Discriminant Analysis Effect Size) analysis results indicate that compared to the RH group, the SH group has significant differences in 1 bacterial family, 2 genera, and 7 species; in contrast, compared to the SH group, the RH group has significant differences in 1 phylum, 2 classes, 2 orders, 1 family, 1 genus, and 1 species. In the SH group, the significantly altered bacterial family is Porphyromonadaceae; the significantly altered genera are Porphyromonas and

**FIGURE 6:**
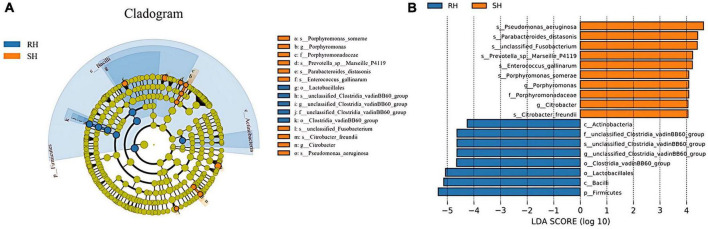
Analysis of the differences in gut microbiota after different surgical approaches. **(A)** LEfSe analysis providing phylum-to-species phylogenetic distribution; **(B)** LDA scores identifying differential entities between the two groups (LDA score ≥ 4. 0).

Citrobacter; the significantly altered species are *Pseudomonas aeruginosa*, *Parabacteroides distasonis*, *Prevotella sp Marseille P4119*, *Enterococcus gallinarum*, unclassified Fusobacterium, *Porphyromonas somerae*, and *Citrobacter freundii*. In the RH group, the significantly altered phylum is Firmicutes; the significantly altered classes are Bacilli and Actinobacteria; the significantly altered orders are Clostridia vadinBB60 group and Lactobacillales; the significantly altered family, genus, and species are unclassified Clostridia vadinBB60 group (Figure 6).

### 3.2 Metabolomic pathway prediction analysis of different surgical approaches

**FIGURE 7:**
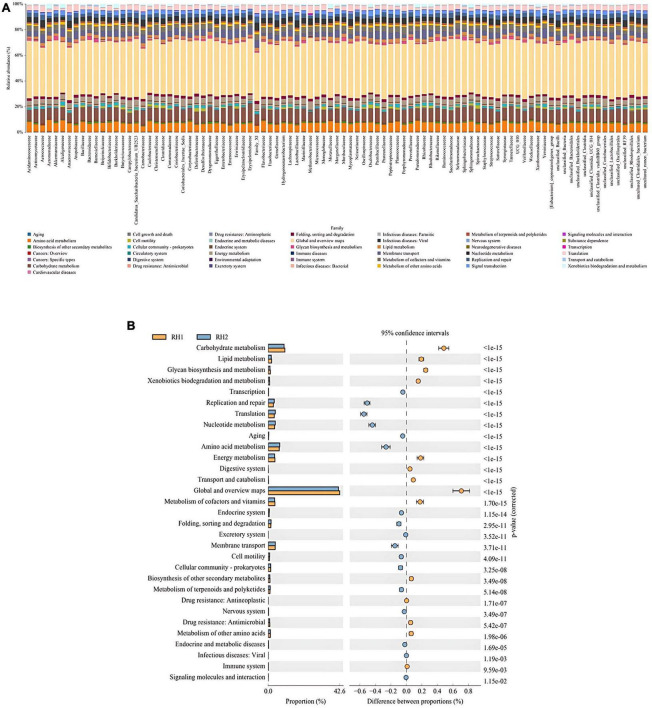
Functional prediction analysis of metabolic pathways of differential biological functions. **(A)** KEGG pathway abundance at the species level; **(B)** differences in metabolism between groups.

To further assess the functionality and role of the host gut microbiota, this study employed the PICRUSt2 software to predict the functional pathways of the 16S rRNA genes of the gut microbiota following two different surgical approaches. The pathway analysis by PICRUSt2 indicated no significant differences between the RH and SH groups (*P* > 0.05). However, upon comparing RH1 and SH1, we found that a total of 39 metabolic pathways exhibited significant changes. Compared between the two groups, 15 pathways were upregulated in the RH1 group (*P* < 0.05), while the remaining 24 pathways were downregulated in the SH1 group (*P* < 0.05). These functional pathways are primarily concentrated in metabolic routes involving amino acid metabolism, glycometabolism, nucleotide metabolism, and carbohydrate metabolism, as shown in Figure 7. The gut microbiota affected by the two distinct surgical approaches participated in multiple metabolic pathways, with the SH1 group activating more metabolic pathways compared to the RH1 group.

## 4 Discussion

Gastric cancer is one of the most common malignant tumors in the digestive system, ranking fifth in incidence and fourth in mortality among the most common malignant tumors worldwide (Sung et al., 2021). The normal gastric mucosa undergoes a progressive sequence of changes from non-atrophic gastritis, atrophic gastritis, intestinal metaplasia, intraepithelial neoplasia, and ultimately to gastric cancer. To date, surgery is still considered the only definitive treatment method. However, surgery can induce stress responses in the patient’s body, including inflammation, ischemia-reperfusion injury (IRI), activation of the sympathetic nervous system, and increased release of cytokines (Chen et al., 2019). In recent years, with the development and widespread clinical application of next-generation sequencing technology, many researchers have found that the gut microbiota plays an important role in the occurrence and development of various diseases, especially in cancer (Abdi et al., 2021; Fernandes et al., 2022; Wong et al., 2023).

In this study, we compared the α-diversity of the gut microbiota postoperatively between the R-Y surgery group and the DTR surgery group, and no significant differences were found, indicating that different surgical approaches do not affect the total number of species, that is, the absolute number of species in the gut microbiota. Regarding the β-diversity of the gut microbiota, we found significant differences between the two groups, indicating that different surgical methods can impact the structure of the gut microbiota. Postoperatively, the species significantly enriched in the DTR surgery group postoperatively include *Pseudomonas aeruginosa*, *Parabacteroides distasonis*, *Enterococcus gallinarum*, *Porphyromonas somerae*, and *Citrobacter freundii*, among which *Pseudomonas aeruginosa*, *Enterococcus gallinarum*, *Porphyromonas somerae*, and *Citrobacter freundii* are conditional pathogens that have significant impacts on the occurrence and progression of cancer, as well as postoperative infections (Caselli et al., 2019; Li et al., 2018; Dziri et al., 2022; Nagre et al., 2022). *Pseudomonas aeruginosa* possesses a high level of antibiotic resistance and can release low molecular weight molecules as chemical signals to regulate the host’s immune response (Miller and Bassler, 2001; Romero et al., 2011), thereby posing a serious threat to the recovery of postoperative patients (Aliaga et al., 2002; Maeda et al., 2012). *Enterococcus gallinarum* is commonly found in pulmonary and biliary infections, leading to symptoms such as fever and respiratory distress in patients post-surgery (Liu et al., 2024; He et al., 2024). In studies related to lung cancer, *Porphyromonas somerae* has been found to be significantly enriched (Yuan et al., 2023), which may be related to postoperative infections that lead to the formation of lung cancer. *Citrobacter freundii* can cause a variety of infections, with urinary tract infections being one of them, leading to symptoms such as frequent urination, urgency, pain during urination, and even hematuria in patients (Huang et al., 2023). *Parabacteroides distasonis* is a beneficial bacterium that can play a protective role in certain diseases (Le et al., 2015; Spinler et al., 2017; Chai et al., 2017). It is widely distributed in the gastrointestinal tracts of humans and animals, and is a Gram-negative, non-spore-forming, rod-shaped strict anaerobe (Rea et al., 2010). It can convert primary bile acids into secondary bile acids and alleviate metabolic disorders by regulating bile acid metabolism (Kang et al., 2019), and moreover, can improve some postoperative symptoms, such as inflammatory bowel disease, by producing or stimulating the host to produce various active metabolites (Zhao et al., 2023).

In the R-Y surgery group, we found an enrichment of Clostridia and Lactobacillales. Clostridia belong to the phylum Firmicutes, are facultative anaerobic bacteria, and mainly consist of beneficial and harmful clostridia, which are very important for the physiological and pathological development of the body. Some are mainly used for the prevention, diagnosis, or treatment of diseases (Miquel et al., 2013; Xie et al., 2016), and some have been proven to be closely related to the occurrence and development of certain human diseases (Spitzer and Ratzan, 1991; Shinha and Hadi, 2015). The Lactobacillales is an important group of probiotics that can synthesize substances such as bacteriocins, organic acids, and polysaccharides, which exhibit activities with antimicrobial, antioxidant, and antitumor properties (Li et al., 2014; Li et al., 2015; Wang et al., 2018). In addition, our analysis also revealed that the R-Y surgery group has a higher F/B (Firmicutes/Bacteroidetes) ratio compared to the DTR surgery group. Firmicutes and Bacteroidetes are the dominant bacterial groups in mammals, accounting for more than 90% of the gut microbiota (Human Microbiome Project Consortium, 2012). Their ratio is commonly used to assess the degree of gut microbial dysbiosis (Zhang et al., 2015), with a higher ratio being more conducive to nutrient absorption and energy storage (Zhang et al., 2021).

In the analysis of the gut microbiota of patients undergoing two different surgical procedures, patients in the DTR group were found to have a higher enrichment of bacteria associated with postoperative infections, which may be related to symptoms such as pulmonary infection, reduced gastrointestinal motility, and gastroesophageal reflux disease. This is consistent with studies conducted in the follow-up of patients after gastric cancer surgery (Li et al., 2024; Peng et al., 2024; Sun et al., 2024). Compared to the DTR group, although the R-Y group had more beneficial bacteria and a smaller amount of pathogenic bacteria detected, it does not necessarily prove that the R-Y surgical approach is superior to the DTR surgical approach. Comparative analyses related to DTR and R-Y surgical methods have shown that the DTR surgical approach is superior to the R-Y surgical approach in terms of short-term nutritional status after surgery and long-term vitamin B12 levels (Qiu et al., 2022). Therefore, when choosing a specific surgical method for gastric cancer patients, a comprehensive comparison and analysis should be made.

This study also has certain limitations. Firstly, there is not a large sample size, which does not adequately reflect the changes in the gut microbiota after surgery. Additionally, no analysis or detection of metabolites was conducted, nor was further verification in animal models, so it is not possible to specifically analyze which metabolites play a role in postoperative infection and the pathways they affect.

## Data Availability

The original contributions presented in the study are publicly available. This data can be found here: https://www.ncbi.nlm.nih.gov/sra, accession number SRP558847.

## Supplementary material

Supplementary Figure 1 Comparison of preoperative and postoperative gut microbiota α-diversity in the DTR surgery group. **(A)** ACE index; **(B)** Chao1 index; **(C)** PD whole tree index; **(D)** Shannon index; **(E)** Simpson index.

Supplementary Figure 2 Comparison of preoperative and postoperative gut microbiota α-diversity in the R-Y surgery group. **(A)** ACE index; **(B)** Chao1 index; **(C)** PD whole tree index; **(D)** Shannon index; **(E)** Simpson index.

Supplementary Figure 3 Analysis of gut microbiota β-diversity pre- and post-surgery in the DTR surgery group. **(A)** PCA analysis; **(B)** PerMANOVA analysis; **(C)** UPGMA clustering tree using weighted UniFrac distances.

Supplementary Figure 4 Analysis of gut microbiota β-diversity pre- and post-surgery in the R-Y surgery group. **(A)** PCA analysis; **(B)** PerMANOVA analysis; **(C)** UPGMA Clustering tree using weighted UniFrac distances.
