# Supplementary figures and images for: The impact of different gastrointestinal reconstruction techniques on gut microbiota after gastric cancer surgery
Source: Front Microbiol. 2025 Jan 24;15:1494049. doi: 10.3389/fmicb.2024.1494049 (PMC11804259; doi:10.3389/fmicb.2024.1494049)

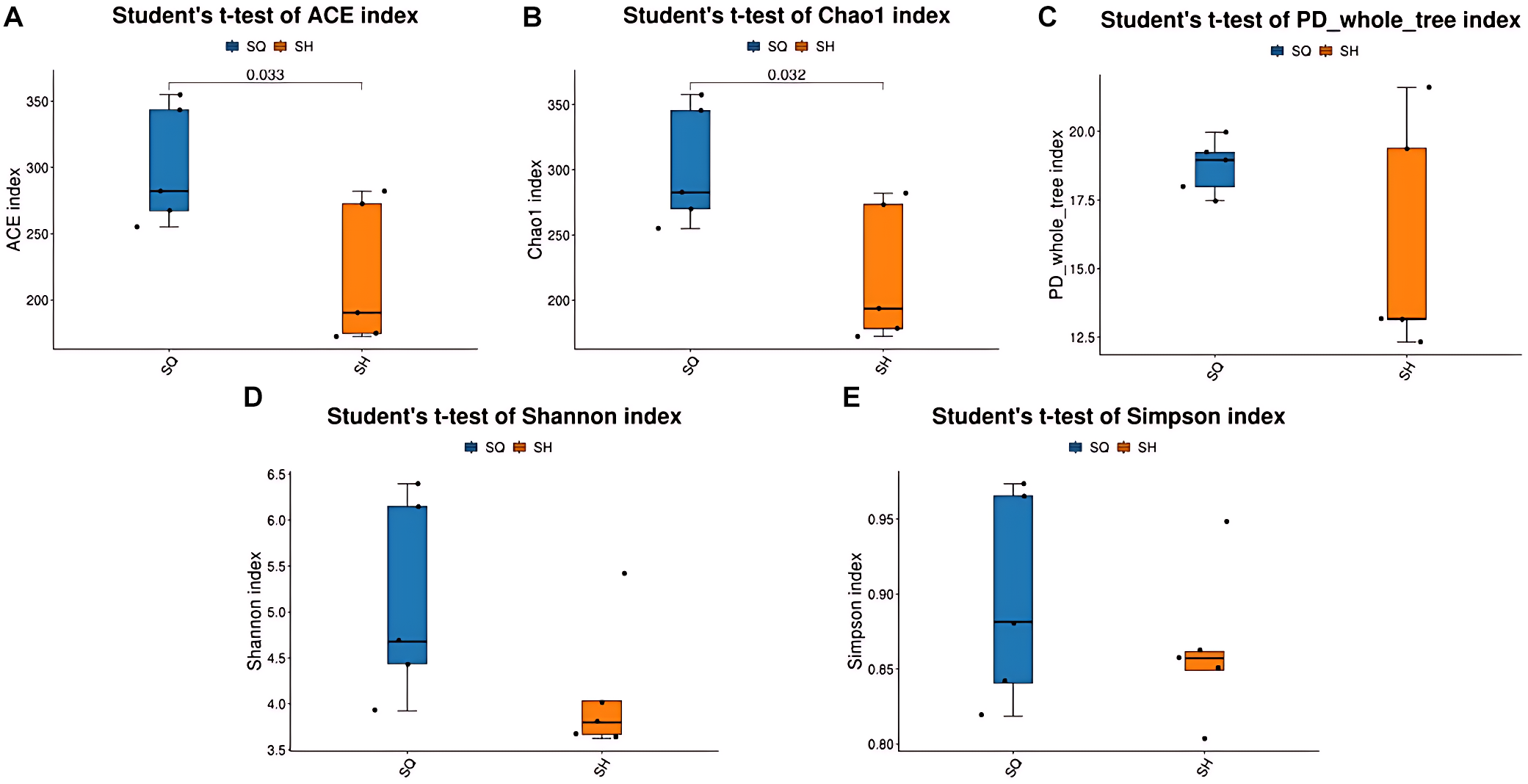

Supplement: Supplementary Figure 1 — Comparison of preoperative and postoperative gut microbiota α-diversity in the DTR surgery group. (A) ACE index; (B) Chao1 index; (C) PD whole tree index; (D) Shannon index; (E) Simpson index. [file Image_1.tif]

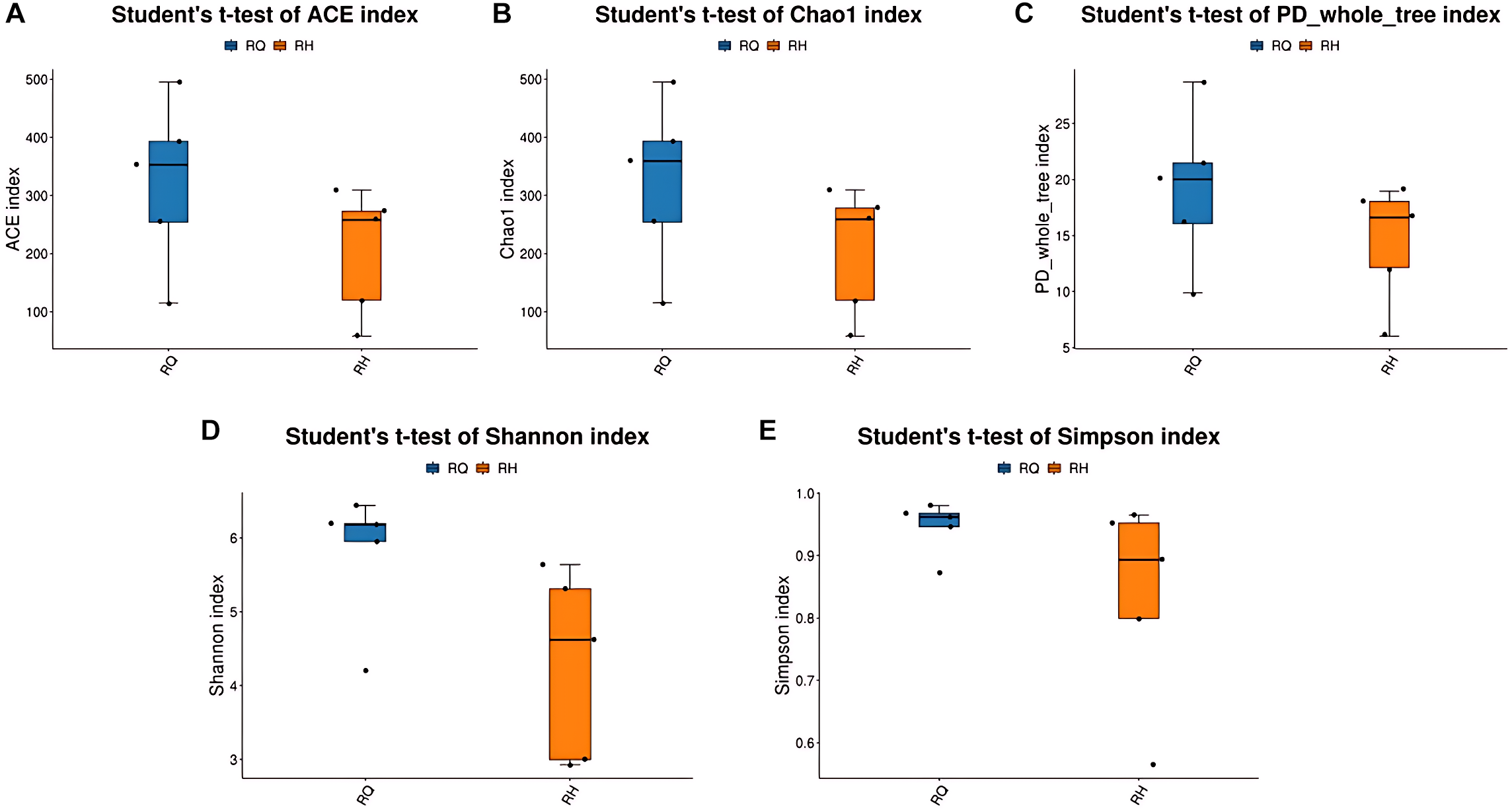

Supplement: Supplementary Figure 2 — Comparison of preoperative and postoperative gut microbiota α-diversity in the R-Y surgery group. (A) ACE index; (B) Chao1 index; (C) PD whole tree index; (D) Shannon index; (E) Simpson index. [file Image_2.tif]

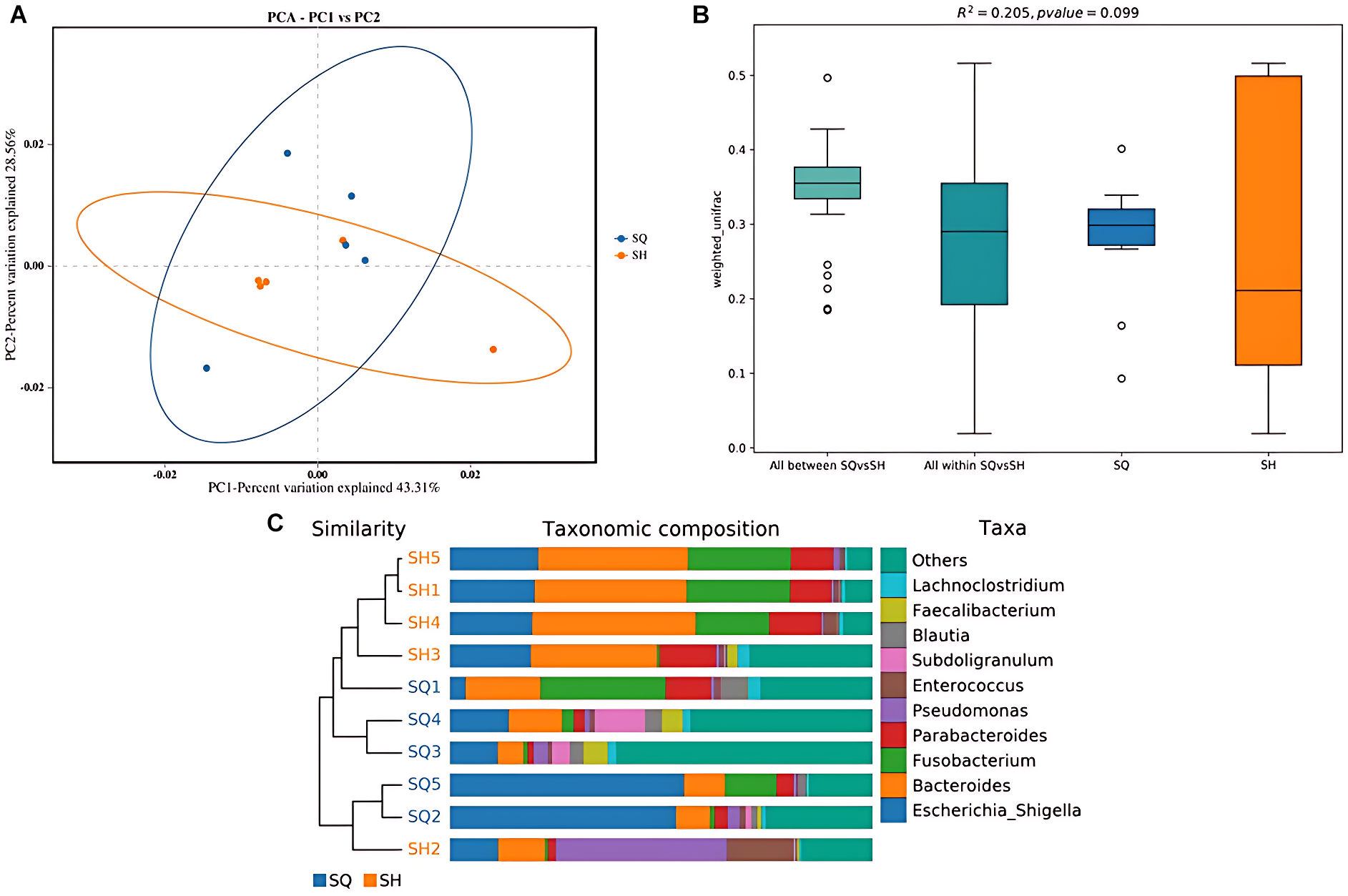

Supplement: Supplementary Figure 3 — Analysis of gut microbiota β-diversity pre- and post-surgery in the DTR surgery group. (A) PCA analysis; (B) PerMANOVA analysis; (C) UPGMA clustering tree using weighted UniFrac distances. [file Image_3.tif]

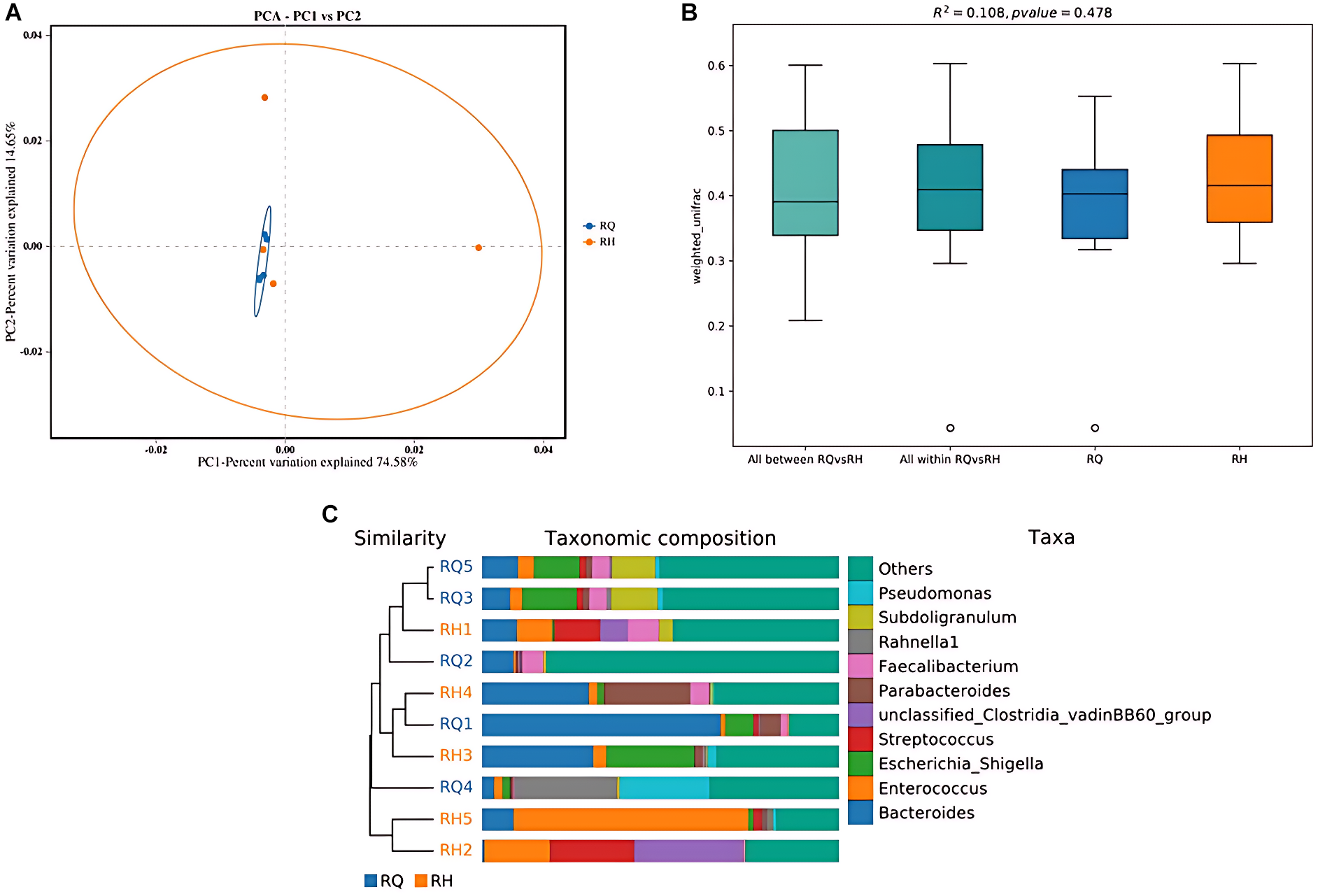

Supplement: Supplementary Figure 4 — Analysis of gut microbiota β-diversity pre- and post-surgery in the R-Y surgery group. (A) PCA analysis; (B) PerMANOVA analysis; (C) UPGMA Clustering tree using weighted UniFrac distances. [file Image_4.tif]
